# Capecitabine and Temozolomide in Neuroendocrine Tumor of Unknown Primary

**DOI:** 10.1155/2018/3519247

**Published:** 2018-05-07

**Authors:** Aman Chauhan, Zainab Farooqui, Le Aundra Murray, Heidi L. Weiss, Zin War Myint, Arun Kumar A. Raajasekar, B. Mark Evers, Susanne Arnold, Lowell Anthony

**Affiliations:** ^1^Division of Medical Oncology, University of Kentucky, Lexington, KY, USA; ^2^Markey Cancer Center, University of Kentucky, Lexington, KY, USA; ^3^School of Medicine, University of Pikeville, Pikeville, KY, USA; ^4^School of Medicine, University of Kentucky, Lexington, KY, USA; ^5^Department of Biostatistics, University of Kentucky, Lexington, KY, USA

## Abstract

Incidence of low grade well-differentiated neuroendocrine tumors (NET) is on the rise. The North American Neuroendocrine Tumor Society estimates that the United States has more than 150,000 gastroenteropancreatic NET patients. About 10% of metastatic NETs can be unknown primary, and due to their rarity, dedicated treatment algorithms and regimens are not defined. Combination of capecitabine and temozolomide (CAPTEM) is one of the systemic treatments used in gastroenteropancreatic NETs. We explored clinical activity of CAPTEM in NET of unknown primary.* Methods*. Retrospective review of NET of unknown primary managed at the University of Kentucky over the past five years (2012–2016).* Result*. 56 patients with NET of unknown primary were identified; 12 patients were treated with CAPTEM. Median progression-free survival on CAPTEM in grade II and grade III NET of unknown primary was 10.8 and 7 months, respectively. Six patients showed reduction in metastatic tumor volume at three-month CT scan. Three patients had stable disease and three patients showed disease progression at the first surveillance scan. Common side-effects were as follows: four patients developed grade II thrombocytopenia, three patients developed grade I lymphocytopenia, and two patients developed hand foot syndrome (grades I and III). Six patients developed grade I fatigue.* Conclusion*. CAPTEM should be considered for grades I and II NET of unknown primary, especially in the case of visceral crisis or bulky disease.

## 1. Introduction

Better diagnostics, recognition, and knowledge of neuroendocrine tumors (NETs) have led to consistent growth in their incidence and prevalence. SEER data suggests a 6-fold increase in the incidence from 1 per 100,000 patients in 1973 to almost 7 per 100,000 patients in 2012 [1]. Despite rising burden of the disease, therapeutic options are limited. Combination of capecitabine and temozolomide (CAPTEM) is one of the chemotherapeutic options for progressive metastatic gastroenteropancreatic neuroendocrine tumors. Data regarding the use of CAPTEM in NETs of unknown primary is lacking. NET of unknown primary accounts for about 10–15% of all NETs [2]. Historically NETs of unknown primary are thought to be relatively aggressive and confer a poor prognosis [3]. We present our single center experience of CAPTEM in this very rare cohort of disease.

## 2. Methods

Patients with neuroendocrine tumor of unknown primary were identified from the Markey Cancer Center's database over a period of five years (2012–2016). IRB approval was obtained for retrospective chart review prior to starting the study. Neuroendocrine tumor of unknown primary was defined by lack of visual evidence of primary tumor on CT scan and or Octreoscan. Neuroendocrine tumors were histopathologically graded based on 2010 WHO classification. Grade 1 was defined as Ki-67 < 2%, grade 2 had Ki-67 index between 2% and 20%, and grade 3 was defined as Ki-67 index more than 20%. Patients treated with CAPTEM regimen were analyzed for radiologic response in the first scan, time to progression on treatment, and toxicity. We used 1500 mg/m^2^ capecitabine and 200 mg/m^2^ temozolomide for treatment of our patients. Capecitabine was administered continuously from days 1 to 14 daily in two divided doses (q 12 hours). Temozolomide was administered on days 10–14 in two divided doses (q12 hours). Eastern cooperative oncology group performance status criteria were utilized to assess the fitness of patient. Grade 0 was defined as fully active and being able to perform all activities without restrictions. Grade 1 was defined as restricted strenuous physical activity but able to carry out light work. Grade 3 was defined as being able to perform limited self-care. Grade 4 was completely disabled and grade 5 was defined as deceased. We used common terminology criteria for adverse events (CTCAE 4.03) to define toxicity. We did not use RECIST criteria to assess radiological response. Radiological response was categorized into progressive disease, stable disease, partial response, and complete response based on descriptive reading of radiological report.

### 2.1. Patient Characteristics

A total of 56 patients with neuroendocrine tumors were identified. Twelve of these patients were treated with capecitabine and temozolomide. The median age of the patients 12 patients was 62 and 6 of them were women and 6 were men. Three patients had an ECOG Performance Status (ECOG PS) of 0 and remaining 9 patients had an ECOG PS of 1. A specific Ki-67 was available in two patients, the solitary grade 1 NET was 1%, and a grade 2 NET was 15%. The remaining patients were graded as I, II, and III as per the current guidelines that classify grade 1 as a Ki-67 of less than 2%, grade 2 between 2 and 20%, and grade 3 as more than 20%. One patient had a grade 1 NET, 7 with grade 2 NET, and 4 with grade 3 NET. Ten patients were classified as well-differentiated NET and two patients were poorly differentiated NET. Three patients had received previous treatments; one of them a grade 2 NET received carboplatin with etoposide and everolimus prior to the use of CAPTEM, the second patient a grade 3 NET received cisplatin with etoposide followed by carboplatin with paclitaxcel, and a third patient with grade 3 NET received carboplatin and etopside. The chromogranin A level was available at the time of starting CAPTEM in 8 of the 12 patients and ranged from 3.8 to 1505 (median 221). The most common sites of metastasis were to liver (10 patients), peritoneum (4 patients), bone (2 patients), brain (1 patient), and the heart (1 patients). Five patients received concomitant somatostatin analogs at the time of treatment with CAPTEM. Eight patients had an Octreoscan performed at the time of start of CAPTEM and four of them demonstrated uptake.

## 3. Results

**Figure 1:**
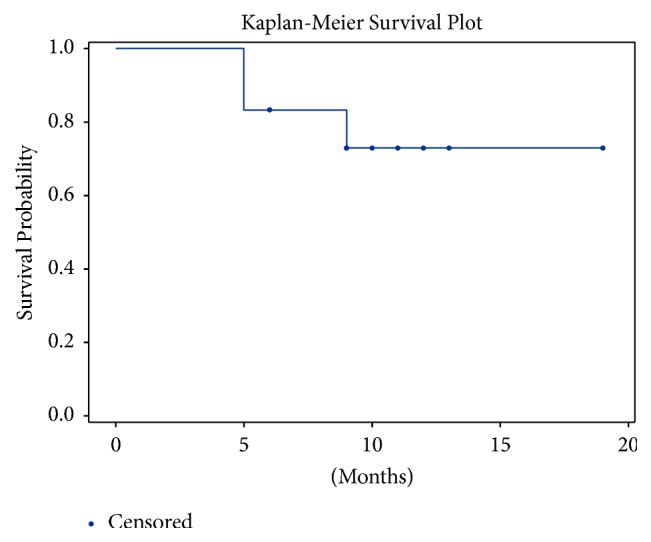
Kaplan-Meier curve depicting progression-free survival while on CAPTEM.

CAPTEM was used as front-line systemic therapy in 9 out of 12 patients. Median progression-free survival (PFS) on CAPTEM in grade II and grade III NET of unknown primary was 10.8 and 7 months, respectively.  Figure 1 shows Kaplan-Meier curve for progression-free survival of NETs of unknown primary on CAPTEM. The median number of cycles received in the entire cohort was 6. The sole grade I NET patient was lost to follow-up after being on treatment for at least 6 months. 6 patients showed reduction in metastatic tumor volume at three-month CT scan. Three patients had stable disease and 3 patients showed disease progression at the first surveillance scan (Table 1). Response rate at three months in grade 2 NET (complete response/partial response) was 57% and response rate in grade 3 NET was 25%. Disease control rate (complete response/partial response/stable disease) at three months in grade 2 NET was 100%.

Following were the rates of common side-effects: Four patients developed grade II thrombocytopenia, three patients developed grade I lymphocytopenia, two patients developed hand and foot syndrome (one each, grades I and III), and six patients developed grade I fatigue (Table 2).

## 4. Discussion

Neuroendocrine tumors are considered an orphan disease due to low incidence, but despite this fact, the prevalence of neuroendocrine tumors is on the rise. The North American Neuroendocrine Tumor Society estimates that there are over 150,000 gastroenteropancreatic neuroendocrine tumor patients in the United States alone. This imbalance between incidence and prevalence is due to indolent history of disease progression. Because of its orphan status, therapeutic clinical trials are few and the treatment options for progressive disease are limited.

CAPTEM is one of the suggested treatment regimens for progressive well-differentiated neuroendocrine tumors. Capecitabine is a prodrug that is converted from fluoropyrimidine to 5-fluorouracil upon oral intake. 5-Fluorouracil has anticancer activity that induces damage to DNA by inhibiting thymidylate synthase [4, 15]. Temozolomide is a lipophilic methylator derived from dacarbazine—an alkylating agent used as a chemotherapy drug. The cytotoxicity of temozolomide is a consequence of guanine methylation which causes apoptosis of cancer cells [5, 16]. Several studies have researched various combinations of capecitabine and temozolomide with other established chemotherapeutic drugs, such as streptozocin as treatment for unresectable NETs. Though streptozocin-based therapy is considered standard of care, the response rates for these combinations have largely ranged from 6% to 42% and displayed considerable toxicity [4, 15, 17], which restricted its use in clinical settings. Capecitabine monotherapy has also been studied and recorded to be well tolerated in patients. It had a 9.68% overall disease control rate, 36.5-month median overall survival, and a 9.9-month median PFS (range: 4.4–36.7 months) [18]. Single agent temozolomide-based therapy has observed a response rate (RR) of 34% for pancreatic neuroendocrine tumors and 2% RR for carcinoid tumors [16].

The rationale for combining capecitabine and temozolomide came from the hypothesis that the effects of capecitabine would enhance NET cell sensitivity to the lipophilic methylator, temozolomide [4, 15]. Simultaneous administration of both drugs on day 1 had insufficient results and substantial toxicity. A regimen of oral intake of capecitabine preceding temozolomide by 9 days was proposed for optimal synergy [15]. This was most likely accredited to the role of O^6^-methylguanine DNA methyltransferase (MGMT)—a DNA repair enzyme. Capecitabine causes depletion of the MGMT gene, which in turn allows the cancer cells to be highly sensitive to temozolomide. Based on the few studies involving CAPTEM, the dosage for capecitabine ranges from 600 mg/m^2^ to 1500 mg/m^2^, averaging at 750 mg/m^2^. Temozolomide doses range from 150 mg/m^2^ to 200 mg/m^2^. Typically, capecitabine is administered on days 1 to 14 and temozolomide on days 10 to 14 every 21 or 28 days.

There is very limited literature that observes the outcomes of CAPTEM as treatment for well-differentiated neuroendocrine tumors.  Table 3 summarizes the current data on CAPTEM in NETs. A recent Phase II study evaluated the efficacy of CAPTEM in NET patients and reported 97% clinical benefit in their study cohorts, which included pancreatic NET, gastrointestinal NET, pituitary gland, and medullary thyroid neuroendocrine tumors. They reported an overall relative risk of 43%, including 11% complete responses, and 54% stable disease rate [6]. Another recent study evaluated the outcome of 65 NET patients treated with CAPTEM. It is the largest study on CAPTEM to date and the data was extracted from the abstract. The majority of study patients (46/65) were pancreatic neuroendocrine tumors (PNETs) and the RR was 47.7%, including 3.1% complete responses, 44.6% partial responses, and 41.5% stable disease. Median PFS was recorded to be 16.1 months [7].

The results from the studies in Table 3 are encouraging and confirm clinical antitumor activity and manageable toxicity. Although data from a Phase III randomized controlled trial regarding use of CAPTEM is lacking, it is a widely accepted treatment for progressive gastroenteropancreatic NET cases that have failed FDA approved front-line therapies. Of note, most of the studies showed a better response rate when CAPTEM was used as front-line therapy.

Current literature on CAPTEM mainly stems from pancreatic neuroendocrine tumor patients [8]. Evidence for the high sensitivity of PNETs to CAPTEM has been attributed to the lower expression of MGMT in PNETs than other NET subtypes. Studies that have compared the outcomes and efficacy of CAPTEM in patients with PNETs versus non-PNETs do show significant overall response rates even in non PNETs, although PNETs are noted to show a more favorable response. Treatment on PNETs exhibited an objective response rate (ORR), disease control rate (DCR), and PFS of 43–70%, 70–97%, and 12–18 months, respectively. Comparatively, treatment on non-PNETs showed an ORR, DCR, and PFS of 33–42%, 56–64%, and 10.5–22 months [15]. Other studies comparing PNETs to non-PNETs found no difference in PFS between the two cohorts, although the data was not statistically significant [4, 9]. An important factor which was noted to affect response rates was timing of CAPTEM. This regimen was found to be less effective in patients previously treated with other targeted therapies. A recent study evaluating CAPTEM as first-line therapy versus subsequent lines reported a median PFS of 15.9 months versus 3.1 months, respectively [9]. CAPTEM's effectiveness with regard to Ki-67 and MGMT expression are currently being researched in clinical studies. The literature shows an exceptionally higher response to CAPTEM when Ki-67 > 5% (ORR = 64%) than when Ki-67 < 5% (ORR = 29%) [19]. However, explicit conclusions cannot be made on the predictive roles of MGMT or Ki-67 without further evidence-based research.

Though various subtypes of NETs have been studied, there is a significant lack of data on the effectiveness of CAPTEM in NET of unknown primary. Only two studies mentioned in Table 3 incorporated NET of unknown primary in their analysis. Peixoto et al. included 3 patients with unknown primary and reported PFS data (12 months) on only one of those three [4]. Spada et al. also included a group of NET of unknown primary along with gastroenteropancreatic subtype [20]. Our cohort is the largest NET of unknown primary treated with CAPTEM. Our results suggest definite clinical activity of CAPTEM in this rare cohort of NETs. Moreover, the ability of CAPTEM to cytoreduce the tumor with a relatively safe side-effect profile makes it a reasonable choice of systemic therapy in progressive NET of unknown primary.

*Limitations*. Because neuroendocrine tumors of unknown primary are a rare disease, the study population was small. In addition, as a retrospective study missing data and a nonrandomized patient population were constraints in the analysis. We did not use RECIST criteria for radiological response assessments.

## 5. Conclusion

CAPTEM shows activity in NET of unknown primary. Current FDA approved treatment options for grade I and grade II GI NETs includes somatostatin analogs and everolimus, both of which are cytostatic and of limited use in case of visceral crisis or bulky disease where disease shrinkage is required. CAPTEM should be considered for grades I and II NET of unknown primary, especially in the case of visceral crisis or bulky disease. While a randomized trial would be optimal to confirm the benefit of CAPTEM in NET of unknown primary, the rarity of these tumors precludes the feasibility of a prospective trial.

## Figures and Tables

**Table 1:** Patient characteristics.

Characteristic	Number
Total patients with NET	56
Total patients treated with CAPTEM	12
Median age	62 years
Male : female ratio	1 : 1
Mean duration of treatment before progression	10.8 months
CAPTEM used as front-line systemic therapy	9 patients
Median chromogranin A	221
Concomitant Somatostatin analogues use	5 patients
Grade I	1
Grade II	7
Grade III	4

**Table 2:** Rates of common side-effects.

Side-effect	Grade	Number
Thrombocytopenia	II	4
Lymphocytopenia	I	3
Hand and foot syndrome	I	1
Hand and foot syndrome	III	1
Fatigue	I	6

**Table 3:** Outcomes and results of CAPTEM.

Primary site, %	Pts (n)	Prior treatment	CAP/TEM dosage (mg/m^2^)	Results (%)	PFS (months)	Observed toxicities, %
Peixoto et al. [4]						
Pancreatic, 48.3 Small bowel, 20.7 Lung, 10.3 Rectum, 6.9 Appendix, 3.4 Unknown, 10.3	29	(i) Octreotide (ii) Local therapy (iii) Chemotherapy (iv) Targeted therapy	Cap: 750 Tem: 200	N/A	4.7	N/A

Fine et al. [5]						
Pancreatic, 39 MEN1, 11 Carcinoids, 22.5 Gastrinoma, 11 Glucagonoma, 5.5 Insulinoma, 11	18	(i) Octreotide (ii) Chemotherapy (iii) Chemoembolization	Cap: 600 Tem: 150–200	CR: 6 PR: 56 SD: 22	14	Thrombocytopenia (grade 3 toxicity), 11 Hand-foot syndrome (grade 2), 5.5 Lymphopenia (grade 1/2), 50 Neutropenia (grade 1/2), 44

Fine et al. [6]						
Pancreatic, 39 Atypical/typical carcinoid, 42.8 Pituitary, 10.7 Thyroid gland, 7	28	(i) Octreotide	Cap: 750 Tem: 150–200	CR: 11 PR: 32 SD: 54 PD: 3	22	Lymphopenia (grade 3/4), 32 Hyperglycemia (grade 3/4), 15 Thrombocytopenia (grade 3/4), 3

Crespo et al. (abstract only) [7]						
Pancreatic, 70.8	65	N/A	N/A	CR: 3 PR: 45 SD: 42	16	Grade 3/4 toxicity, 13.8 Thrombocytopenia, 10.8 Neutropenia, 7.7

Strosberg et al. [8]						
Pancreatic, 100	30	(i) Octreotide (ii) Local therapy	Cap: 750 Tem: 200	PR: 70 SD: 27 PD: 3	18	Grade 3/4 toxicity, 12 Hand-foot syndrome

Ramirez et al. [9]						
Pancreatic, 52 Small bowel, 31 Lung, 10 Rectal, 7	29	(i) Cytoreduction (ii) Targeted therapy (iii) Radionuclide therapy (iv) Chemotherapy	Cap: 750 Tem: 200	PR: 17 SD: 48 PD: 34	12	Thrombocytopenia grade 1 (3), grade 2 (3), grade 3 (1) Lymphocytopenia grade 1 (3), grade 2 (9), grade 3 (3) Neutropenia (grade 4), 3 Hand-foot syndrome grade 1 (6), grade 2 (3)

Spada et al. [10]						
Pancreatic, 55 GI & unknown, 24 Lung, 21	58	(i) Octreotide (ii) Chemotherapy	Cap: 1,500 Tem: 150–200	PR: 22 SD: 52 PD: 23	13	Thrombocytopenia (grade 3/4)

Fine et al. [11]						
Metastatic NETs	10	(i) Octreotide (ii) Chemotherapy	Cap: 750 Tem: 150–200	CR: 16 PR: 34 SD: 50	NA	Hand-foot syndrome (grade 3), one case.

Saif et al. [12]						
Pancreatic, 100	7	(i) Octreotide (ii) Local therapy (iii) Chemotherapy	Cap: 1,000 Tem: 200	PR: 43 SD: 28 PD: 29	12	Thrombocytopenia (grade 3), one case. Fatigue (grade 3), one case Neutropenia (grade 1/2) Hand-foot syndrome (grade 1/2)

Abbasi et al. [13]						
Pancreatic Rectum Colon Stomach	21	(i) Octreotide (ii) Chemotherapy (iii) Local therapy	Cap: 600 Tem: 50–200	PR: 57 SD: 23 PD: 20	17	No grade 4 toxicity

Ramirez et al. [14]						
Pancreatic, 50 Small bowel, 30 Lung, 13 Rectum, 7	30	N/A	N/A	PR: 33 SD: 40 PD: 27	11	Cytopenia, 25 Hand-foot syndrome, 35

N/A: not available, CR: complete response, PR: partial response, SD: stable disease, PD: progressive disease, Cap: capecitabine, and Tem: temozolomide.
